# Hospice Caregivers’ Perception of Family and Non-Family Social Support and Stress over Time: Associations with Reports of General Support

**DOI:** 10.3390/ijerph20065009

**Published:** 2023-03-12

**Authors:** Maija Reblin, Djin L. Tay, Eli Iacob, Kristin G. Cloyes, Megan C. Thomas Hebdon, Lee Ellington

**Affiliations:** 1 Department of Family Medicine, Larner College of Medicine, University of Vermont, Burlington, VT 05405, USA; 2 College of Nursing, University of Utah, Salt Lake City, UT 84112, USA; 3 School of Nursing, Oregon Health & Sciences University, Portland, OR 97239, USA; 4 School of Nursing, University of Texas at Austin, Austin, TX 78712, USA

**Keywords:** caregiver, social support, home hospice, oncology, longitudinal, interpersonal relationship

## Abstract

Social support has been identified as a key factor to protect wellbeing for home hospice cancer caregivers. However, few studies have assessed social support over time in this context, and measures of support are often limited to general assessments of perceived support. Our goal was to (1) describe change in cancer home hospice caregivers’ social support over time during care and into bereavement and (2) explore the impact of perceived stress and support from family and non-family members on caregivers’ perceived general social support. We conducted a secondary analysis of longitudinal prospective questionnaire data. Forty caregivers completed measures of general perceived support, family and non-family support and stress during hospice enrollment and 2 and 6 months post the patient’s death. Linear mixed models were used to determine change in support over time and the contribution of specific support/stress ratings to general support assessments. Caregivers overall had moderate and stable levels of social support over time, though there was significant variation between and within individuals. Family and non-family support and stress from family predicted general perceptions of social support, while no effects were found for non-family stress. This work suggests a need for more specific measures of support and stress, and the need for research to focus on improving baseline levels of caregiver perceived support.

## 1. Introduction

Family caregivers provide essential support to cancer patients throughout their trajectory, from diagnosis to treatment to end of life [1]. While providing care can be meaningful, the burden of caregiving responsibilities can negatively impact the caregiver’s quality of life [2]. Caregiving responsibilities and burden can intensify at the end of life [3], especially upon enrollment to home hospice. About 48% of Medicare decedents received hospice care in 2020, with a median length of stay of 97 days [4]. During this time in the hospice, family members take the lead in providing care with support of an interdisciplinary hospice care team. Because of the physical and emotional burden often required to provide care at the end of life and through bereavement, hospice family caregivers need support to maintain their own health and well-being.

Social support has been identified as a key protective factor against adverse health outcomes [5,6]. Theoretical models suggest that social support confers emotional and tangible resources than can be used to cope with stress [7,8]. For example, support can include information or help making decisions, tangible assistance in providing care or managing other responsibilities, or reassurance or emotional validation. Despite the dynamic nature of cancer caregiving, the majority of research focused on social support and caregiving is cross-sectional [6] and little prospective longitudinal research exists to identify the pattern of social support over time during and after home hospice care. Within this limited body of research, findings are mixed regarding the stability of caregiver perceived support early in the care trajectory [9,10], and research on bereavement indicates that perception of social support generally increases over time [11,12]. As such, support may be less available during the period of end-of-life caregiving, when it may be most needed. In addition, research suggests that the number of family members available to offer support may decrease in the first four weeks of hospice by a small but significant amount [13].

In addition to a lack of longitudinal assessment, the measures used to assess social support often are quite general, capturing only a broad assessment of perceived support [6]. While family and friends can provide important support, many caregivers report conflict in their relationships or unhelpful support that can contribute to burden and stress [14,15]. In fact, support and stress can co-occur within the same relationship [16]. For example, a friend could provide important help with household tasks but may also offer unwanted or misinformed advice [17]. Additionally, there can be differences between an individual’s general perceived support, which encompasses the overall perception that general support will be available if needed (i.e., *someone* will help) and the perceived support from specific members of one’s social network (i.e., *my sister* will help).

### Objective

The objective of this secondary analysis is to (1) describe changes in cancer home hospice caregivers’ perceived general social support over caregiving and bereavement and (2) explore the impact of perceived stress and support from family and non-family members on caregivers’ perceived general social support.

## 2. Materials and Methods

This is a secondary data analysis of a multi-site longitudinal prospective observational study of family caregivers of cancer home hospice patients conducted between 2017 and 2020. Additional information about the primary study is available elsewhere [18].

### 2.1. Sample and Setting

We conducted a secondary data analysis with data from hospice caregivers of patients with a primary diagnosis of cancer. Eligible primary caregivers were aged 18 years and above, English speaking, and provided in-home hospice care to the patient. Family and non-family caregivers were eligible; however, parental caregivers were not eligible due to the difference in the caregiving experience for a dying child. Caregivers were recruited from hospice agencies in four states across the US.

This study was approved by the University of Utah Institutional Review Board in February 2016 (IRB#00088662). All participants provided written informed consent.

### 2.2. Measures

We collected demographic information from caregivers including age, gender, race, ethnicity, and whether caregivers cohabitated with the patient at baseline. Social support measures were assessed at baseline, at the 2nd month, and the 6th month of bereavement.

#### 2.2.1. General Perceived Social Support

General perceived social support was assessed with a 4-item version of the Medical Outcomes Study Social Support (MOSS) survey [19,20] at all timepoints. The MOSS responses are measured on a 5-point Likert scale ranging from 0-none of the time to 4-all of the time. Scores are summed and transformed to a scale ranging from 0–100 with higher social support resp. Higher scores indicating higher perceived social support. The internal consistency of the MOSS for this sample was Cronbach’s α = 0.87.

#### 2.2.2. Family and Non-Family Support and Stress

The Duke Social Support and Stress Scale (DUSOCS) was administered to assess family and non-family sources of support and stress [21,22] at all timepoints. Caregivers rated the level of support and stress provided via family relationships (spouse or significant other, children or grandchildren, parents or grandparents, siblings, other blood relatives, and other relatives by marriage), and non-family relationships (neighbors, co-workers, church members, and other friends). Responses ranged from “none or there is no such person”, “low”, and “high”. Scores were summed in accordance with the DUSOCS user guide resulting in scaled scores from 0 (low)–100 (high) [23].

### 2.3. Analyses

Descriptive statistics were calculated for all key measures at baseline enrollment in hospice, and 2 months and 6 months after patients’ death. An intraclass correlation coefficient (ICC) was computed to evaluate the proportion of variance associated with general perceived social support (using the MOSS) accounted for by the repeated measures over time. The ICC was 0.61, indicating within-person correlation in general perceived social support across time points.

A series of linear mixed models (LMMs) using a restricted maximum likelihood estimator and diagonal covariance matrix were used to estimate parameters accounting for the interdependence of the repeated measures. Fixed effects included time (treated as categorical with baseline as reference category), age, caregiver/patient cohabitation (reference category: not cohabitating), gender (reference category: female), and the specific DUSOCS subscales of family support, family stress, non-family support, and non-family stress. Random effects included intercept variance and variance at each time point. Models were examined for normally distributed residuals based on plots and non-significant Shapiro–Wilk tests and plots of predicted values and residuals with models demonstrating reasonable assumption of homoskedasticity.

## 3. Results

Data from 40 caregivers who completed questionnaires at all time points were used (of 102 who consented and completed baseline data [18]). Caregiver demographics are shown in Table 1.

Table 2 shows descriptive statistics for the outcomes at the three timepoints for the 40 caregivers included in the analysis. Caregivers overall had moderate levels of general perceived social support (Ms = 63.07–65.94) across the three timepoints compared to scores in the general population in prior work (M = 70.1; SD = 24.2 [20]), and reported highest levels of social support from family members. While aggregate scores demonstrated high stability over time, there was significant variation across individuals.

Pearson correlations across all measurement timepoints found that general perceived support was positively correlated with family support (r = 0.496, *p* < 0.001), negatively correlated with family stress (r = −0.280, *p* = 0.002) and nonfamily stress (r = −0.237, *p* = 0.009), but not associated with non-family support (r = 0.065, *p* = 0.484).

Mixed effects models with random intercept examined demographic characteristics and time only (Model 1) with the added value of inclusion of the DUSOCS subscales of family and non-family social support and stress (Model 2). Model 1 of general perceived social support (MOSS measure) included time, co-residence, gender, and age. As shown in Table 3, there were no significant fixed main effects for time compared to baseline for 2 months (β = −2.511, SE = 3.173, CI [−8.921, 3.900]) or 6 months (β = −4.541, SE = 3.655, CI [11.869, 2.787]). Furthermore, no association with gender (β = −6.827, SE = 6.887, CI [−20.821, 7.167]), caregiver/patient cohabitation (β = 13.568, SE = 7.480, CI [−1.631, 28.767]), or age (β = 0.041, SE = 0.244, CI [−0.455, 0.537]) with MOSS measures.

Model 2 included baseline characteristics with the DUSOCS subscales for both family and non-family support and stress subscales. Controlling for demographic covariates, there were positive associations for MOSS with Family Support (β = 0.335, SE = 0.101, CI [0.133, 0.536]), and Non-Family Support (β = 0.277, SE = 0.095, CI [0.088, 0.466]), and negative association with Family stress (β = −0.276, SE = 0.121, CI [−0.515, −0.036]). Non-family stress was not significantly associated with MOSS (β = −0.147, SE = 0.164, CI [−0.473, 0.179]). Inclusion of the DUSOCS subscales (Model 2) significantly improved model fit compared to Model 1 (χ^2^ (4) = 14.075, *p* = 0.007). As indicated in Table 3, both models had significant random effects variance for intercept and at each timepoint, suggesting high variability in MOSS score between subjects and across time. Interaction effects of subscale by time were explored and all were non-significant. We therefore retained the more parsimonious models.

A 95% confidence interval lower bound (LB) and upper bound (UB) χ^2^ test examining change in log likelihood with addition of DUSOCS stress and support subscales for family and non-family. Random effects: σ^2^ intercept variance; Time baseline, 2 months, and 6 months repeated measures variance

## 4. Discussion

Social support is a key factor in protecting against depression and reduced quality of life, and as such has been identified as particularly important for those providing care in cancer home hospice and into bereavement [2,24]. However, the literature is limited by the lack of specificity of social support measures, which are often focused on general perceived support, and not prospectively assessed over time. This study describes both broad and specific measures of support in an end-of-life cancer caregiving context over time. Our findings, similar to work in broader relationship contexts [25,26], suggests that adding additional information about more specific aspects of social interactions adds predictive value compared to more general measures of support.

Our findings suggest that hospice family caregivers on average report levels of support just below average for the general population, which remain relatively stable over time. This may be the result of selection bias, such that caregivers who elect to engage in home hospice—and research—are likely to have more resources. However, other research suggests that while many cancer caregivers report at least moderate levels of general perceived support in late stages of end-of-life caregiving and in bereavement, many also endorse specific unmet support needs, including needs focused on the patient’s condition as well as needs relevant to caregiver well-being and relationships [27,28,29].

Our work emphasizes that both family and non-family support is important for overall perceptions of support for family caregivers. In part, this may point at the role of close non-family relationships, including “chosen family,” such that friends and neighbors can become as or more important than biological family for well-being [30,31,32]. Our findings also indicate the importance of the broader social network in caregiving [33,34]. For example, coworkers and neighbors can offer seemingly small but important support, such as flexibility in scheduling meetings or helping with yard maintenance, that frees up caregiver time and mental load. Research on non-biological/legal family support and the importance of broader social networks in caregiving is limited but growing [35,36,37]; future work can elucidate how caregivers can best leverage this support.

In addition, we found preliminary evidence that stress from family, but not non-family sources was influential in affecting general perceptions of social support. This may be due to the ability for individuals to more easily manage the frequency and context of interactions with non-family members to limit situations where stress or conflict may occur [16]. For example, coworkers can often avoid discussing conflicting opinions about religion, but this may be more challenging among family members. The stability of reports of social support, also seen in other longitudinal research in advanced cancer caregivers [38], suggests that helping caregivers to establish good social support networks early in the care trajectory—and ideally, before taking on caregiving responsibilities—is a key target to protect caregiver well-being over time. Further, our analysis suggests that reducing family stress and resolving family conflicts may be a more important upstream target than promoting additional support [39].

At the same time, our findings also indicate wide variability in general support, support and stress from family, and support and stress from non-family both between and within individuals over time. Given the complexity of social support perception, there are likely many drivers of this variability. One potential intrapersonal explanation that may be highlighted in our work due to its longitudinal nature is changing caregiver expectations and availability of support [40,41]. For example, cancer caregivers may expect a particular level of support based on their previous experiences of support. However, support needs and availability may differ at cancer diagnosis, cancer recurrence, hospice enrollment, and in bereavement; because each of these represents a new, unique stage, caregivers may be adjusting their perception of supports based on different frames of reference of what came before. Additional explanations for variability in perceived support may include interpersonal or social network factors, such as shifting relationships among non-caregiving family members, contextual factors, such as external stressors from work or other care responsibilities, or systemic factors, such as changes to eligibility or availability of formal resources that increase the pressure on informal network members.

### 4.1. Limitations

While this paper contributes an assessment of the stability of caregivers’ social support surrounding end of life, the findings should be interpreted with caution. The primary limitation of this study is the relatively small, homogenous sample of caregivers who provided complete data. The difficulties in recruiting and retaining hospice caregivers in longitudinal research is well documented [42,43]. Although we were unable to determine reasons for non-participation, it is possible that caregivers who experienced greatest strain or negative wellbeing, or more socially isolated caregivers chose not to enroll or complete study measures. Although our predictive analyses may not be adequately powered, our results from this secondary analysis suggest there may be some robust effects and our findings may be used as preliminary data to guide future work. Future studies should focus on recruitment of a larger, more diverse sample of cancer caregivers to account for the influence of other covariates. Similarly, research focused on more frequent ecological assessments of expected, perceived, and received support across the care trajectory would contribute to explaining key mechanisms of functional support and ultimately could contribute to a better understanding of caregiver well-being.

### 4.2. Clinical Implications

Clinicians should consider the variability in the sources of important support for family caregivers; specifically, clinicians should be mindful that non-family support may be particularly helpful. Because of the overall variability between and within caregivers over time, attention should be paid to each caregiver’s individual situation to ensure timely referral to programs to augment informal support with formal resources, such as support groups, respite, or other services, as cancer caregivers are a population at-risk for poorer physical health outcomes and deal with substantial emotional tolls of caregiving particularly in hospice [44,45]. Clinicians should also be aware that family stress can impact the perception of caregivers’ overall support; programs to help manage this stress may be important to include in end-of-life care.

## 5. Conclusions

We find that cancer hospice family caregivers’ reported levels of support are generally moderate and stable over time, though there is high between and within-caregiver variability. Reports of general support are likely driven more specifically by perceptions of high family support, though perceptions of non-family support and family stress may also play a key role. This work can guide future research to identify other key drivers of perceptions of support, as well as interventions focused on improving baseline levels of caregiver perceived support.

## Figures and Tables

**Table 1:** Demographics of Caregivers (n = 40).

Characteristic	
Age	M (SD)
Mean ± SD	59.64 ± 14.12
Gender	N (%)
Male	11 (27.50)
Female	29 (72.50)
Race	
White/Caucasian	35 (87.50)
Asian	1 (2.50)
American Indian, Alaska Native, Aleut, Eskimo	1 (2.50)
Black or African American	1 (2.50)
Multiple races	2 (5.00)
Ethnicity	
Non-Hispanic	36 (90.00)
Hispanic	4 (10.00)
Marital Status	
Married/committed relationship	34 (85.00)
Single (never married)	2 (5.00)
Separated/Divorced	3 (7.50)
Widow or widower	1 (2.50)
Highest Education	
High School or Equivalent or Less	5 (12.50)
Some College or Vocational School	16 (40.00)
College Graduate	8 (20.00)
Some Graduate or Professional School	2 (5.00)
Graduate or Professional Degree	9 (22.50)
Employment	
No	17 (42.50)
Part Time	8 (20.00)
Full Time	14 (35.00)
Missing	1 (2.50)
Perceived Adequacy of Financial Situation	
Not very good	7 (17.50)
Comfortable	24 (60.00)
More than adequate	7 (17.50)
Missing	2 (5.00)
Co-residence with Patient	
Yes	29 (72.50)
No	11 (27.50)
Relationship to Patient	
Spouse/Partner	21 (52.50)
Sibling	2 (5.00)
Adult Child	16 (40.00)
Friend/Other	1 (2.50)

**Table 2:** Descriptive statistics at Baseline, 2 Month Bereavement, and 6-month Bereavement (n = 40).

		Baseline		2 Months		6 Months	
Outcome	Scale	M (SD)	Min–Max	M (SD)	Min–Max	M (SD)	Min–Max
MOSS: General Perceived Support	0–100	63.07 (24.51)	0–100	63.49 (23.88)	12.5–100	65.94 (22.29)	25–100
DUSOCS: Family Support	0–100	48.04 (19.94)	7.14–85.71	47.14 (19.66)	14.29–100	47.32 (18.28)	14.29–78.57
DUSOCS: Non-Family Support	0–100	38.75 (25.03)	0–90	41.25 (18.70)	10–80	39.00 (20.23)	0–90
DUSOCS: Family Stress	0–100	18.57 (17.18)	0–57.14	19.82 (16.21)	0–57.14	20.36 (17.46)	0–57.14
DUSOCS: Non-Family Stress	0–100	5.75 (13.75)	0–60.00	4.25 (7.81)	0–30	6.00 (12.15)	0–50

**Table 3:** Mixed effects modeling examining Family and Non-Family support and stress on General Social Support.

	Model 1: MOSS on Demographics					Model 2: MOSS on DUSOCS Stress/Support Subscales				
				95% Confidence					95% Confidence	
	β	SE	Sig	LB	UB	β	SE	Sig	LB	UB
Intercept	67.966	17.459	<0.001	32.527	103.405	49.645	18.478	0.010	12.493	86.797
Base (ref)										
Time (2 months after death)	−2.511	3.173	0.433	−8.921	3.900	−1.862	3.090	0.551	−8.133	4.410
Time (6 months after death)	−4.541	3.655	0.220	−11.869	2.787	−4.922	3.002	0.108	−10.968	1.123
Live separate	13.568	7.480	0.078	−1.631	28.767	10.273	6.981	0.151	−3.928	24.473
Live together (ref)										
Female	−6.827	6.887	0.329	−20.821	7.167	−3.540	6.297	0.578	−16.382	9.303
Male (ref)										
Age	0.041	0.244	0.868	−0.455	0.537	−0.035	0.232	0.881	−0.505	0.435
DUSOCS Family Support	X					0.335	0.101	0.001	0.133	0.536
DUSOCS Non-Family support	X					0.277	0.095	0.004	0.088	0.466
DUSOCS Family Stress	X					−0.276	0.121	0.025	−0.515	−0.036
DUSOCS Non-Family Stress	X					−0.147	0.164	0.373	−0.473	0.179
Number of Parameters	10					14				
−2 log likelihood	991.735					977.660				
Model 1 v Model 2 (χ^2^, *p* value)	Ref					(14.075, 0.007)				
Random Effects	Est	SE	Sig	LB	UB	Est	SE	Sig	LB	UB
σ^2^	305.167	91.208	<0.001	169.875	548.207	244.387	78.570	0.002	130.142	458.922
Time (baseline)	112.727	49.716	0.023	47.492	267.569	176.277	61.511	0.004	88.956	349.316
Time (2 months)	241.153	71.306	<0.001	135.083	430.511	147.527	56.402	0.009	69.734	312.106
Time (6 months)	279.922	80.955	<0.001	158.805	493.413	194.876	65.454	0.003	100.893	376.403

## Data Availability

Data is available upon reasonable request to the senior author.
